# A Scoping Review of the Health of Conflict-Induced Internally Displaced Women in Africa

**DOI:** 10.3390/ijerph17041280

**Published:** 2020-02-17

**Authors:** Oluwakemi C. Amodu, Magdalena S. Richter, Bukola O. Salami

**Affiliations:** 1 Faculty of Nursing, University of Alberta, Level 3—Edmonton Clinic Health Academy, Edmonton, AB T6G 1C9, Canada; bukola.salami@ualberta.ca; 2 Faculty of Nursing and Global Nursing Office, University of Alberta, Edmonton AB T6G 1C9, Canada; solina.richter@ualberta.ca

**Keywords:** internally displaced women, scoping review, women’s health, Africa, health

## Abstract

Armed conflict and internal displacement of persons create new health challenges for women in Africa. To outline the research literature on this population, we conducted a review of studies exploring the health of internally displaced persons (IDP) women in Africa. In collaboration with a health research librarian and a review team, a search strategy was designed that identified 31 primary research studies with relevant evidence. Studies on the health of displaced women have been conducted in South- Central Africa, including Democratic Republic of Congo (DRC); and in Eastern, East central Africa, and Western Africa, including Eritrea, Uganda, and Sudan, Côte d’Ivoire, and Nigeria. We identified violence, mental health, sexual and reproductive health, and malaria and as key health areas to explore, and observed that socioeconomic power shifts play a crucial role in predisposing women to challenges in all four categories. Access to reproductive health services was influenced by knowledge, geographical proximity to health services, spousal consent, and affordability of care. As well, numerous factors affect the mental health of internally displaced women in Africa: excessive care-giving responsibilities, lack of financial and family support to help them cope, sustained experiences of violence, psychological distress, family dysfunction, and men’s chronic alcoholism. National and regional governments must recommit to institutional restructuring and improved funding allocation to culturally appropriate health interventions for displaced women.

## 1. Introduction

Conflict and internal displacement are underexplored issues, but they are the significant social determinants of health for persons around the world [1]. It is estimated that 60% of preventable maternal deaths, 53% of under-five deaths, and 45% of neo-natal deaths take place in settings of conflict and displacement [2]. Women and children make up around 80% of internally displaced populations, and international humanitarian agencies have recognized the special needs of women who are fleeing with children [3]. Displaced women face special challenges in accessing healthcare, which is linked to rising death rates from maternal complications of unplanned pregnancies and unattended childbirth in the displacement settlements where they have sought refuge [3,4]. International agreements exist that afford international migrants and refugees’ special protection and entitlements, but unfortunately they do not provide the same protections to all internally displaced persons (IDPs) [5]. Global attention to the health needs of IDPs has similar gaps. International policy frameworks have been disproportionately centered on health for migrants and refugees to developed countries, since richer destination countries are increasingly mandated to recognize their ethical responsibility towards health and human rights protection for immigrants and refugees [6]. IDPs, however, have not crossed international borders; therefore, since they remain within the binding institutions of their home counties, their health access is subject to the provisions made by the presiding government, which is often still in the throes of sustained political unrest [7].

Notably, Africa hosts one-third of the world’s forcibly displaced persons [8]. UN peace-keeping and the UN Commission for Humanitarian Affairs have played an active and important role in maintaining security in war-torn African states, restoring political order, and addressing the acute hunger and shelter needs of those displaced people [8,9]. Yet, these interventions have downstream effects; further, they do not address the long-term needs for empowering communities to manage the social and economic consequences of the conflict and displacement [10].

The United Nations Population Fund (UNFPA) identifies that displacement is a gender issue: the face of displacement is female [11]. The grey literature and academic literature both agree that women are disproportionately affected by the negative consequences of conflict-induced displacement, yet little is known about its long-term impact on them and their health [12,13,14,15]. Similarly, women’s health issues in the context of displacement in Africa have been underexplored [4,16,17], likely related to the fact that no human rights protocols exist to protect these women’s rights to access health care. Research in the field of displacement has skewed toward sexual violence and prevention of unplanned pregnancies, a result of the gender-based violence women experience. Conflict-related sexual violence is a well-established tactic of terrorism in many African countries and around the world [18]. Moreover, most displacement crises in Africa happen in environments with significant power differentials and deep-rooted inequality, in which the conditions are already unfavorable to women and girls’ sexual freedoms [19]. An example is the well-known 2014 Chibok abduction and subsequent sexual violations of schoolgirls in Nigeria, an apparent strategy to vehemently oppose western education for girls [20].

Over the last decade, through cluster interventions, the UN has made remarkable inroads in addressing basic humanitarian needs and ameliorating the protection and assistance gaps for IDPs [7]. Nevertheless, Olajumoke Jacob-Haliso has critiqued the United Nations High Commissioner for Refugees on their approach to forced displacement in Africa, describing it as a blanket approach that obscures African women’s unique marginal experiences in various refugee settings [21]. In particular, displaced women carry an unequal burden of care because of a gendered responsibility toward caring for children and the elderly. In circumstances of conflict, women often are the last to flee, are usually fleeing with children, and may have to undertake in transactional sex to provide food for themselves and their children. This increases the chances of exposure to physical and mental health problems, infectious diseases, and to sexual violation [1,3].

Grey literature reports and research provide evidence that women are affected negatively by conflict-induced displacement, but no peer review study has reviewed the existing evidence on health problems affecting women who are internally displaced, even though this population remain one of the most vulnerable groups of migrants. The International Organization for Migration has identified that attaining the United Nations Sustainable Development Goal “to leave no one behind,” ultimately entails creating a more inclusive and equitable society: this suggests that marginalized and disempowered persons should be the center of development activities [22], which would certainly include internally displaced women. Planning effective programs to address their needs is necessary, but such efforts must depend on evidence-based understandings. The current review discusses all the studies related to the health of internally displaced women in Africa to identify research gaps and areas where policy interventions should be focused.

## 2. Materials and Methods

A comprehensive literature search was performed using the keywords internal* AND displace* AND Africa* in major health databases. Specifically, with the support of a librarian, we reviewed and retrieved peer-reviewed articles written in English from these databases: CINAHL (n = 347), Ovid MEDLINE (n = 112), EMBASE (n = 334), PsycINFO (n = 138), and Global Health (n = 247). The search was updated between 2017 and 2020, when five additional studies were identified.

### Preferred Reporting Items for Systematic Reviews and Meta-Analyses (PRISMA)

**Figure 1:**
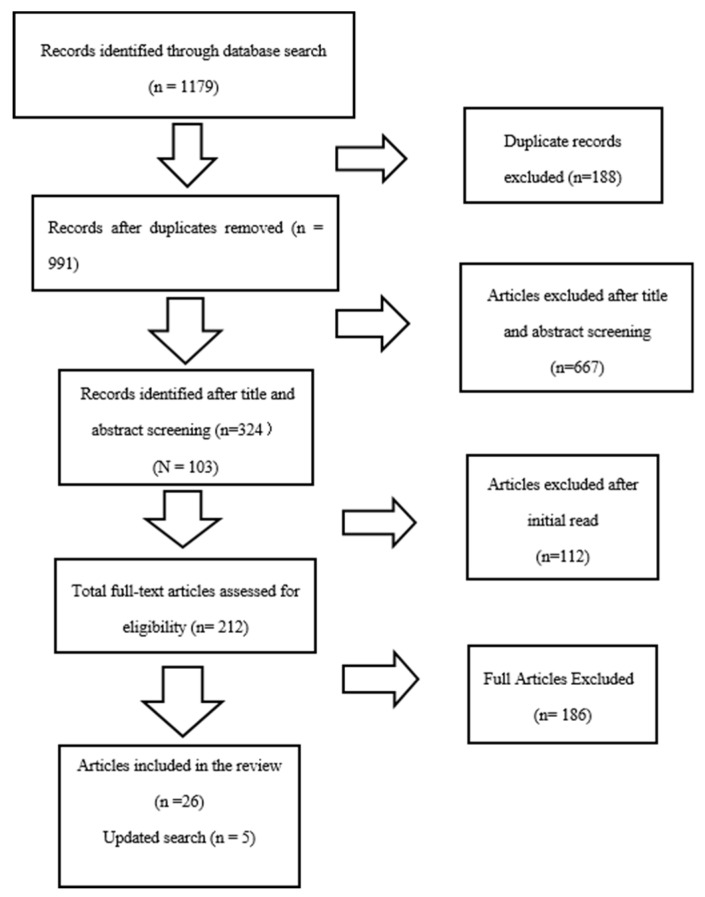
PRISMA flow diagram.

The review process was guided by the PRISMA guidelines for systematic reviews [23]. Two reviewers screened the papers. The most experienced researcher gave feedback about the discrepancies between the reviewers’ approaches. The first database search was completed in March of 2017, with the results updated in January of 2020. There were no date limits. The PRISMA flow diagram of the review search is shown in Figure 1; a total of 1179 records were retrieved from the database search. After duplicates were deleted, 991 articles remained. Article screening was performed using RefWorks Citation Manager, with the following inclusion criteria: research articles published in English that focus on the health of internally displaced women in Africa. Articles with any of the following exclusion criteria were not reviewed:

- A focus on women who migrated outside of their country’s borders, who were displaced by a natural disaster, or who are economic migrants;
- A focus on instrument testing or the piloting of questionnaires;
- Lacking a methodology;
- Merely literature reviews or discussion papers;
- Grey literature, reports, websites, and graduate theses;
- Published in a language other than English.

A two-step process was followed for study screening: the first step involved screening by title and abstract; the second step involved screening by an initial read of the full text. Titles and abstracts were screened against the inclusion criteria. After the title and abstract screening, 324 were identified, which was further reduced to 212 after the initial reading. Then, after the final assessment for eligibility by all members of the team, 26 articles, with an added 5 updated retrievals, were included in the extraction table. (see Figure 1).

## 3. Results

The 31 articles were charted in Microsoft Word using the study characteristics of author, purpose of study, design, country/setting of research, sample size, and study results (see Table 1). The locations of these studies were Sudan (n = 7), Uganda (n = 11), Eritrea (n = 2), Cote d’Ivoire (n = 1), Angola (n = 1), Democratic Republic of Congo (DRC) (n = 3), Sudan and DRC (n = 1), Sudan, Uganda, and DRC (n = 1), Burundi and Uganda (n = 1), and Nigeria (n = 3). The methodology used in the studies varied, but many included quantitative surveys (n = 15). The studies did not hinge on any theoretical standpoint. Diverse methods were used in the studies, as follows: cross-sectional survey (n = 15), mixed methods (n = 5), focus group(s) (n = 2), semi-structured interviews (n = 4), qualitative psychological autopsy method (n = 2), neighborhood methodology (n = 1), descriptive method comprising a focus group and semi-structured interviews (n = 1), and a clinic record review (n = 1). We categorized results according to several subtopics related to aspects of the health of internally displaced women, as follows: violence (n = 7), mental health (n = 10), sexual and reproductive health outcomes (n = 9), and malaria (n = 5).

### 3.1. Violence

Our review shows that violence against displaced women by intimate partners and by others is a socially and psychologically damaging practice in conflict-affected communities in Africa. In countries like northern Uganda, Ethiopia, and DRC, over two decades of conflict and gender-based violations have served to reinforce a culture of disregard, and thus impunity, for diverse forms of violence [24,25,26]. Prolonged displacement brings about community apathy, because social cohesion and trust are eroded by war [27]; additionally, domestic violence becomes normalized, families live in chaotic, overcrowded settlements without any form of privacy. In other words, private incidents of violence become known to neighbors and, being a frequent occurrence, the community becomes numb. This applies, for example, to the issue of marital rape—even when this has happened and neighbors know of it, they no longer intervene [24].

The practice of domestic violence affects the mental health of women, children’s orientation to sexuality, and the value of parental authority. Marital rape also results in children having premature sexual awareness, contributing to girls losing interest in education because of early sexual engagements with the opposite sex [24,28]. Gender role dysfunctions, threats to masculinity, and women’s non-conformity to the expected subordinate roles were significant factors making them more vulnerable to domestic violence [25,26].

Eight studies explored domestic violence, in which marital rape had variable meanings. In northern Uganda, Stark et al. [25] explored violence among 204 households and identified that about 50% of respondents had experienced intimate partner violence in their marriages. Cardoso et al.’s work expanded on the causes of marital rape, associating its prevalence with social and structural changes to the environment of the displaced [27]. The study, conducted in Cote d’Ivoire in West Africa, identifies that urban poverty—with its high male unemployment, food insecurity, financial stress, and cramped housing— played a role in women’s experiences with intimate partner violence. Women talked about their own stress and anxiety over financial constraints, as well as losing interest in being sexually intimate with their husbands [27]. A cross-sectional study, conducted in Kampala, Uganda, found that 53% of women reported experiencing violence in Kampala’s urban settlement, with married participants having higher odds of intimate partner violence than single ones [29].

Marital rape and abuse were weapons for men to enact masculinity and familial control; they were sometimes portrayed by men as signifying “commitment.” In Uganda, men pointed to the ubiquity of violence and its approval by religious leaders, thereby justifying the act and capitalizing on the victim-shaming culture to perpetuate marital abuse [24]. Women reported that their husbands publicly boasted about the rape, talking about it at drinking places [24]. Excessive social drinking practices pervade masculine culture in Uganda and Nigeria, as a masculine coping strategy for war-related distress, which only exacerbates familial tensions [28]. A study conducted in Nigeria on social constructions of sexuality and pleasure in displacement settings also affirmed acceptance of sexual violence. Women did not perceive their sexually aggressive husbands as violent, but rather used words like “shameless” and “impatient” to describe this behavior [28]. Women in the study placed more emphasis on the moral impact the sexual violence would have on their children’s sexuality. Also, children in these crowded living conditions may see their parents having sex, which happens more openly in such contexts. The concern here is with the unhealthy sexualization of IDP children because of the lack of sexual privacy and their parents’ loss of authority, leading to moral decadence among youths.

Stark et al. [26] conducted a study with a large sample size of over 1788 young women, identifying that in DRC and Ethiopia, violence against displaced women was perpetrated in public spaces, such as at water collection points, sports fields, forests, schools, markets, and roads—by military soldiers and police, as well as the host community. Oladeji also identified sexual violence-related pregnancies among women who have been freed from insurgencies and were now living in conflict-affected north east Nigeria. Of the sexual violence cases that were identified, 0.6% resulted in pregnancies. All women who attended the health clinic at the camp requested to terminate these pregnancies at the initial contact; service providers suspected they moved out of the camps into host communities to achieve this outcome [30].

Economic difficulty leads married and single women to engage in transactional sexual practices. Financial insecurity and unemployment of spouses, for example, as well as a lack of social support led some women to have random sexual engagements to get access to money to provide for their families [24,27]. This behavior has an impact on the sexual and reproductive health outcomes for displaced women.

### 3.2. Mental Health

Mental health was explored in ten studies [31,32,33,34,35,36,37,38,39,40], which revealed that violence and mental health were intricately linked. Suicide and its precipitating circumstances were explored in two qualitative psychological autopsy interviews in northern Uganda among women in a post-conflict context [38,39]. The traumatizing effects of prolonged conflict, post-conflict gender role shifts, loss of traditional systems of social support, and socioeconomic challenges associated with displacement converged to create mental health problems for displaced persons [32]. In South Darfur, six registered IDP camps were surveyed in Nyala District. The study identified a 31% prevalence of major depression among women [37]. In Northeast Africa, research showed that displacement had significantly more negative effects on women compared to men. Women in IDP camps had lower scores on sense of coherence scales, and scores worsened with the experience of serial displacement [31,32].

Almedom et al. found that the cultural and economic context of communities affected women’s ability to adapt to the psychological effect of displacement, including outcomes for sense of coherence. For example, certain communities in Eritrea that had been used to transhumance herdsmen in subsistence farming had a better sense of coherence than those communities of a different occupation [32]. Another study by Roberts et al. measured rates of post-traumatic stress disorder (PTSD) and depression among IDPs, investigating the associated demographic and trauma exposure risk factors through a cross-sectional survey conducted in northern Uganda [40]. This study showed that while men reported higher exposure to traumatic events than women, men reported lower levels of mental distress [40]. For instance, 49% of women compared with 71% of men had experienced eight or more traumatic events. Yet, women were twice as likely as men to exhibit symptoms of PTSD and over four times as likely as men to exhibit symptoms of depression [40]. This study also noted that women exhibited emotional symptoms, not psychotic ones. Hamid and Musa (2010) investigated the effects of the Darfur crisis on the mental health of internally displaced women—in particular, the traumatic events and resulting living conditions inside camps for IDPs in Darfur, Sudan. Results showed that 72% of the participants were classified as having PTSD and symptoms of general distress, such as anxiety and hyper-arousal [33].

Women’s mental health was affected in diverse ways for many reasons. Women in Africa are traditionally committed to caring for their children, but keeping the marital union intact is also primarily seen as the woman’s responsibility, and thus it is a priority. Men, on another hand, feel responsible for financial security of the household [32]. Internally displaced women experience a shift in gender roles creates psychosomatic distress in women because of the added financial responsibility to provide shelter, food, and security [40]. Married participants were more distressed and more anxious—with each person’s psychological health issues being a direct reflection of their partner’s social dysfunction [33,38].

Following conflict and displacement, women bear the responsibility for the family’s livelihood, in addition to their reproductive role—for example, in cases where the men’s movements outside the camps were restricted and the external food aid was limited [27], or when men had lost their jobs, which was a common occurrence [25,29]. Despite the changed reproductive roles, husbands wanted to remain in control of the family’s resources and expected the same sexual dominance and authority over their wives. However, the women were not prepared to take on the role of primary breadwinner but remain the subordinate partner. As a result, fights could ensue as women tried to restrict or control their husbands’ expenditures [38,39]. Men’s quest to reestablish their masculinity, in the face of its perceived loss, led to them spending resources on alcohol, leisure activities, and extramarital affairs [38].

Women’s attempts to fight for their rights—mainly through resisting sexual advances, which is perceived as a cultural transgression resulted in men seeking to marry new wives. This added to the women’s experiences of being abandoned. Worse still, while married to new wives, some men continued to be abusive to their first wife, acts which triggered suicide attempts for these first wives. Traditionally, these cultures are polygamous, and mothers-in-law would be supportive in cases where a man takes on a new wife; she would orient the new wife to the responsibilities of the home. In one of the cases, the mother-in-law had died, so that the senior wives were forced to take on the role of teaching the junior wife, which creates further stressors [39].

These unconventional stressful experiences were summarized into two broad ideas, identified in two studies: no control in life and no care from family [36,39]. Another study categorized these stressors into three groups: (1) the individual or psychoemotional factors, such as deaths of partners and children, and hardships of life in the camp; (2) the meso-system level risk, resulting from the husband’s decreased participation in family responsibilities, such as farming, caring for children, and financial contributions, with additional stressors at this level being limited access to social and community supports system; and (3) the exo-system level stressors, including decreasing community safety, increased gender-based violence and decreased respect from children [35,36].

### 3.3. Sexual and Reproductive Health

Nine studies explored reproductive health as their key focus, with five focused on family planning [41,42,43,44,45], two focused on the impact of education on the use of reproductive health services [46,47], one concentrated on the effect of armed conflict on access to health services, access to rights, and women’s perceptions of sexual and reproductive health with research conducted in Burundi and northern Uganda [48], and one explored HIV infection among displaced women [49]. Kinyinda [34] and Kim et al. [38], who primarily focused on mental health, also looked at the effect of reproductive health issues on the mental health of displaced women.

Chi et al.’s descriptive and explanatory qualitative study explored the perceptions of displaced women in northern Uganda about the effects of armed conflict on maternal and reproductive health services and outcomes [48]. Their findings reveal that the main mechanisms through which conflict led to poor access and quality of maternal and reproductive health services varied across the IDP sites. Attacks on health facilities and looting of medical supplies in both IDP sites was one reason. As well, other factors intervened: the targeted killing of health personnel, favoritism in the provision of health care in Burundi, and abduction of health providers in northern Uganda [48].

Sexual exploitation in IDP camps predisposes women to sexually transmitted infections including HIV [34,49]. Women living in IDP camps in DRC were shown to have twice the rates of HIV compared to host populations, which is associated with conflict-related sexual violence, and unprotected sex between displaced and non-displaced persons [49].

Women’s limited access to sexual and reproductive health rights was influenced by several factors, including low income status, cultural views, and spousal disapproval [38]. Women’s access to rights also relied on their knowledge and understanding of their rights. For instance, many women did not consider that they had any rights to refuse sex, or to not be physically battered by their husbands [38]. Of 1240 women surveyed, 70% stated that in their marriages, non-consensual sexual intercourse was a normal occurrence, and contraceptive use was disapproved of by their husbands [38].

Adam and Adam et al. conducted research at the same site in Sudan. They explored the impact that home visits/interpersonal communication for maternal education made on Sudanese displaced women’s decisions about where to give birth. They were able to provide this education to 87% of women in their study, linking this to a greater preference for a medical facility for their deliveries (versus choosing a home birth). Those who choose home-births, despite the education, did so for several reasons, such as traditional significance around a home-birth, grandmothers’ advice, husbands’ preferences, and fear of doctors, avoiding surgical intervention, and not having enough time to go to a facility [46].

Maternal health education at home was associated with a 43% reduction in home-based delivery performed by traditional birth attendants in the conflict-affected setting of Darfur, Sudan [46,47]. Adam et al. found that providing a woman with education and free services are two important predictors of facility births and use of reproductive health services, including antenatal and postnatal services [47]. Among all women investigated, hospital delivery improved from 25.9% to 63.1% following a mass education campaign. There were also significant associations between receiving home visits and awareness of tetanus vaccinations and postnatal care visits in the follow-up phases. Interpersonal communication and mass education campaigns where community health workers disseminated information by home/shelter visits, clinic sessions, and public meetings improved the uptake of reproductive health services [47].

Unmet needs for contraceptives was another area of reproductive health explored in three studies. A study examining family planning knowledge and use among women in camps in DRC identified that 65 out of 155 women surveyed reported having an unintended pregnancy mostly because of extramarital affairs or casual sexual relationships [41]. Most women reported that they had received information on contraception and had a moderate knowledge of modern contraceptive methods. Among Congolese IDP women, 84% reported having information on contraception, of whom 50% received information during antenatal care. Of this number, only a few women had used contraceptives. Contraceptive knowledge among female camp residents was moderate, however actual usage was low, predominantly for lack of interest, although additional reason given were insufficient knowledge, religious reasons, and partner refusal [41]. More than 40% of women had experienced an unplanned pregnancy, with a history of abortion associated with non-use of contraceptives [41].

One study in Kassala, Eastern Sudan between 1 July 2012 and 31 July 2012 explored the unmet need for contraceptives. Among 812 married women surveyed, the total demand for contraceptives was 71%. The rates of unwanted and unplanned pregnancies and births were 13% and 16% respectively [42]. A mixed methods study conducted in Angola between April and October 2001 with 7090 among women aged 15 to 49 years of age showed that economic status was a major barrier to accessing a health clinic for health care services [43].

In Sudan, Uganda, and the DRC, current use of modern contraceptive methods among women was less than 4% in four program areas: West and South Darfur, Southern Sudan, and Eastern Congo. Oral contraceptive pills were the most widely used method in every site except Eastern Congo, where condoms were most popular [44]. Where health centers offered oral contraceptives, facilities were ill equipped to offer all the mandated methods of family planning because of a lack of funding for such programming [44]. Some women also felt that there was unfairness in facility services, in that they were being provided with contraceptives but not with reproductive and maternal health services. 

### 3.4. Malaria

The high burden of malaria was a health concern identified in four studies, for which there are limited interventions and controls. Obol et al. conducted a study exploring misconceptions about malaria treatment among internally displaced pregnant women [50]. They found that the role of vector transmission of malaria was poorly understood by women in northern Uganda. Some women thought that cold food, playing in the rain, cold weather, and eating mangos could cause malaria [50].

Draebel et al. identified Plamodium falciparum to be the most common parasite species detected among Nuer Sudanese displaced women [51]. In this malaria study, conducted in 2013, Dræbel et al. identified that primary school attendance was a stronger predictor for use of malaria risk reduction measures than any of the other selected background characteristics. This signifies that an individual’s educational level need not be very advanced to affect their practices of malaria prevention and treatment. School attendance was also significantly associated with insecticide-treated net ownership [51]. For fever caused by malaria in pregnancy, about 65% of displaced women sought treatment within the first 24 h [51]. In South Sudan, Draebel et al.’s 2014 study also found that health centers were a last resort for treatment for malaria [52]. They carried out a qualitative study to explore lay perceptions of malaria and process of treatment among 30 resettled pregnant women. The women preferred to pursue treatments in the following order: home remedies, traditional healers, and self-medication with medicines from vendors or supernatural healers [52]. The women did not clearly link fever as a symptom of malaria; rather, illness if considered to potentially be malaria when it is accompanied by other signs like neurosis and dizziness.

Obol et al. conducted a cross-sectional study in northern Uganda in 2013 to establish the prevalence of, and factors associated with, insecticide-treated net (ITN) use among pregnant women in post-conflict IDP camps of the Gulu district [53]. ITN usage was low (35%) among pregnant women; factors that promoted such usage included antenatal visits, ITN awareness, and a willingness to purchase ITNs. Factors that impeded its usage included the hours of travel necessary to reach a health center and being single, widowed, or divorced [53]. Brooks et al. identified that women in DRC rarely complied with ITN use to prevent malaria except where it concerned their fetuses [54]; thus, presenting information at antenatal health centers about ITNs and their use in preventing malaria was an effective way to increase compliance. Women who did not attend antenatal visits would not have knowledge about the usefulness of bednets, since they would not be exposed to the routine information sessions held at these sites [54]. Another factor that made use of bednets difficult was the space constraints in camp tents, where IDPs slept on mats, making it impossible to tuck away the bednets. Further, the muddy environment easily soiled bednets, but washing them also posed a challenge given the shortage of water in the camps. Food insecurity compelled some women to sell their nets to the host communities [54].

## 4. Discussion

As noted, we categorized the studies into four subtopics. Violence, mental health, sexual and reproductive health, and malaria. Seven studies had findings centered on violence perpetrated by both intimate partners, armed forces, and the general community, ten studies explored mental health, and nine studies explored reproductive health. Violence in post-conflict Africa took different forms depending on the context of the settlement—in particular, whether it was rural or urban. Intimate partner violence was a major finding of this review, including aggressive sexual expectations from men, and dysfunctional masculinity related to progressively being relocated from one region to another without gainful employment opportunities.

Gendered power differences shifted constantly; women’s new roles as economic providers seemed to especially threaten masculine authority. The loss of community and extended family supports served to strain marital relations further, so that domestic violence and other types of violence were connected, and divisions between public and private life in camps blurred. The privacy of the marital environment was non-existent because of the overcrowded tent housing systems. Neighboring families could see and hear what was going on in other people’s home. Violence in intimate spaces was therefore not really intimate, but rather, public—exacerbated by widespread communal violence that served to sanction it. The normalization of violence against women in public spaces was facilitated by public shaming, and further institutionalized because security forces and police forces were also seen to openly violate women. Finally, pastors—who women might have reasonably seen as supportive figures or public figures they could turn to for help—were also documented to be abusive to their own spouses.

The lack of sexual inhibition on the part of men, associated with their traditional masculine feelings of entitlements, along with chronic alcoholism, led to open sexual relations between couples. Children became exposed to these kinds of adult interactions, contributing to diminishing respect for parental authority. As a result, children of the displaced begin to inappropriately imitate adult sexual behaviors at an early age, leading to the degeneration of societal mores and sexual decency. Young people are participating in a sexual economy as a means of survival and married women are turning to sexual labor in a desperate effort to meet the needs of food security for their families. Transactional sex has hence become a norm in post-conflict societies in Africa.

Mental health was explored in ten studies, especially as it pertains to the emotion-centered burden of care and family responsibilities that women shoulder, which led them experience the brunt of family disintegration in a more severe way. The psychological impact of sustained trauma from within the home and from outsiders, the persistent fear of the unknown, the loss of loved ones and livelihoods, housing issues, and lack of family support or community connection created a serious strain on women’s mental health. Further, when familial support systems become eroded during times of social conflict, post-partum stress can take a particular toll and advance to become full blown depression.

Obviously, mental health is increasingly becoming an issue of concern among African’s forcibly displaced people [55]. War and conflict have eroded collective resilience in African communities, which is the main strength of African societies. But gender differences are an important consideration here also, and understanding gender-specific aspects of displacement and health can inform effective long-term mental health service programs to alleviate traumatic disorders [33,56]. For example, a study in Ethiopia evaluated gender as a category that influences coping with trauma, finding that men reported a significantly different experience of traumatic life events related to displacement and perceived social support, when compared to women [57]. In this study, women reported higher emotion-oriented coping whereas men reported higher task-oriented coping. This supports findings from a study among displaced Zimbabweans in South Africa, which identified that pre- and post-migration stress, as well as poor mental health, are related to PTSD for women but not for men. Women’s traumas were more attributable to higher incidences of harassment from the police during the pre-displacement period, while men reported threat-to-life, being hungry, and not having a place to live as their key concerns after displacement [58].

With respect to reproductive health, poor awareness of relevant information was not only found among women; health providers are also inadequately knowledgeable about the guidelines for reproductive health interventions in displacement [46,47]. Religious, cultural, and financial factors also play a role in attitudes toward contraceptive usage among African women [41,42,43,44,45]. Existing studies conducted among displaced adolescents in Africa support this, demonstrating that misconceptions about contraceptives cause adolescents to refrain from using them [59]. Additionally, the culture of home delivery is a common practice in traditional Africa which may also indirectly affect utilization of contraception. The review showed that home visitation and education had a huge impact on encouraging more women to choose a hospital-based delivery, and many women obtain knowledge about contraceptives from antenatal care associated with this birth choice.

However, in general, women have a fair knowledge of contraceptives irrespective of education levels. The challenge with actual uptake relates to cultural norms and expectation for women to have multiple births. The husband’s educational status and position on family planning are also very significant factors determining uptake of reproductive health services. Another aspect of reproductive health is HIV prevalence. Studies examined this issue as it related to sexual violence and casual sex, observing that treatment was difficult to access for displaced people in rural settings. Armed conflict have been shown to expose women to both sexual violations and HIV [48,49].

We identified that malaria in pregnancy was a common illness among displaced women because of the open shelter housing. Moreover, this review showed that many women had misconceptions about malaria management and the effect of malaria on the growing fetus [50]. In managing fever, women sought traditional healers as the first line of treatment. While insecticide-treated nets are being distributed by aid agencies, some women had to sell them in order to provide food for their household [54].

Primitive knowledge on how malaria can be controlled, such as beliefs in herbal treatments and self-medication, were found to be issues preventing prompt treatment [54]. Treatment was usually sought upon the recognition of fever (“loup” in Nuer, which translates to “hotness”); however, the link between fever and malaria, (“juay lieth pouny” in Nuer or “illness of heated/perspiring body”) was not always instantly recognized. Two women (2/30: 7%) identified malarial fever by its cyclical characteristics, but they also stated that only fever accompanied by signs and symptoms of complicated malaria—described as “confusion,” “madness” or “dizziness”—could be identified as malaria [52].

Pregnant women understood the risk differently, with some misconceptions. They expressed phrases like a malaria infection could cause the fetus to “suffer,” become “uncomfortable,” “restless,” or “irritable,” or could cause the fetus to become “anemic.” They also made statements such as “the child is born with spots”; “the mother’s womb will swell up”; or “the child does not move for two to three months” [52]. This suggests that self-diagnosis is a common practice among displaced women, and further suggests that other infectious diseases and HIV, which may present with cyclical fever or malaria-like symptoms, may be wrongly diagnosed as malaria. About 24% of women waited for two to three days before seeking treatment.

The 2030 Agenda for Sustainable Development contains a number of targets related to reproductive health. Specifically, target 3.7 calls for ensuring universal access to sexual and reproductive health care services, including family planning, information, and education, and the integration of reproductive health into national strategies and programs by 2030 [60]. These interventions—in addition to the prevention of mother-to-child transmission (PMTCT), treatment for sexually transmitted infections, vaginal injuries, and fistula management, post-abortion care, safe abortions, interventions for the prevention of sexual violence, and comprehensive clinical management of rape—all need to be explored as equally important components of reproductive health in displacement as outlined in the Minimum Initial Service Package MISP [2]. This review showed that research has been done on the health of displaced women in parts of Africa. Nevertheless, sex-disaggregated information on internal migration in Africa as it pertains to women’s health is scarce [59].

Seven studies employed a qualitative approach, with single methods such as focus group discussion and semi-structured interviews, to explore aspects of the health of displaced women. The drawback of the study methodologies includes a lack of in-depth theoretical analysis of the descriptive accounts of the women. As well, the studies tended to use a qualitative method for data collection but then applied a quantitative method of analysis. This has implications for the kinds of hypothetical associations that can be drawn from most studies, as well as on the causal relationships that can be discerned, particularly with single-method studies.

Most studies were not grounded in any theoretical assumption related to women’s cultural environment, language, and social vulnerability. This was the case with studies examining the unmet needs for contraceptives, which were poorly estimated because of language barriers [43]. As well, a theoretical framework was absent in studies that used surveys to explore women’s STIs, sexual behavior, and gender-based violence, with the conceivable inaccuracy of self-reports of STI symptoms [49]. Shortcomings of findings drawn from self-reporting is related to the inability to validate actual incidence with any clinic information, since health clinics did not have activity logs of clinic visits and kept poor kept health care records and had no antenatal records or vaccination cards [29].

A methodological mismatch was found with studies exploring topics like domestic violence through focus group discussion [11,27]. Domestic violence is a serious problem affecting displaced women, yet cultural beliefs in many African societies and patriarchal culture influence women’s ability to report being violated. Thus, focus group and verbally administered survey methods used to explore topics like intimate partner violence among displaced women may have been subject to social desirability bias [27]. Given the stigmatization of intimate partner violence in the context of African culture, there is no guarantee that participants being asked to speak in a group would be able to give a true account of their experience as survivors or as perpetrators of violence or to share their personal opinion on the matter. This casts the conclusions of these study in some doubt—at least in terms of the reported amount and degree of incidents, even if the qualitative data of the women’s lived experiences and interpretations remain meaningful.

Studies that explored maternal health access often looked at intervention methods, such as studying the impact of home visits on the awareness and uptake of maternal and reproductive health services [46,49]. These studies had no control groups, and therefore it is impossible to theorize the causal relationship between the interventions and changes in the measured outcome indicators in the follow-up survey. One study used a purely qualitative approach (including a descriptive and explanatory qualitative study with semi-structured in-depth interviews and focus group discussions) to explore perceptions of the effects of armed conflict on maternal and reproductive health services and outcomes [48]. The target group was NGOs and local health service providers. The affected displaced women were not interviewed on their reproductive health access.

Another concern in several studies was the researchers’ inadequate consideration for the impact trauma has on the cognitive abilities of displaced women and how this affected the data on management of related mental health issues. Considering that displaced women have been through trauma at different points in the past, their ability to recall previous experiences can be distorted, especially when the current experience is being reported through a structured survey with closed-ended questions. Gender considerations also need to be accounted for in trauma-related studies because women may withhold certain sensitive traumatic events in their past because of the interviewer’s gender (that is, they may find it too hard to share their details with a male interviewer, for example among Hausa communities of northern Nigeria where women cannot share reproductive health information with men) [30].

## 5. Conclusions

This scoping review identified major health concerns among internally displaced women in Africa. Evidently, the available studies on this subject are heterogeneous, yet rich in the description about the various health concerns affecting these women. From the review, it is clear that health issues related to intimate partner abuse, poor access to contraceptives and professional birthing support, and a prevalence of malaria are priority concerns. These concerns can all be linked one way or another with the socioeconomic impact of war, and the changes in gender role expectations between husbands and wives. This is further complicated by the overall fragility of these regions and the attending precarity and housing problems. These African communities are also in need of health facilities, which depends in part on the needed humanitarian aid to rebuild health facilities that have been destroyed during conflicts in the various regions. Our review also identified the gap in research on abortion care, and post-natal outcomes for women who choose to have home- births versus those who access hospital settings for delivery.

This review has useful implications. Building of health facilities, treatment of malaria, and provision of HIV diagnosis and treatments, which the UNHCR cluster strategies already advocates for is an urgent need in African IDP camps. However, the responsibility to bring long-lasting recovery and health access to these populations remains the responsibility of governments in Africa. For decades now, African states have struggled with the issue of internal displacement and the need to advance the rights and healthcare access for displaced women through implementing the Kampala convention [16]. The Kampala policy has, in itself, no specific guidelines on how institutions should implement IDP policies. However, international humanitarian actors must proceed carefully if they want to bring about real changes in conflict-affected countries and thereby improving the lives of internally displaced women. They have an obligation to respect the sovereignty of these African nations, even while they seek to implement policies that will have positive health outcomes. Moreover, Africa cannot continue to depend on these international policy instruments and depleting UN humanitarian aid to transform the status quo in Africa.

African governments need a renewed commitment towards internally displaced persons. Constitutional amendments, especially, must incorporate the health service needs of displaced people, given the many vulnerable people in this population in need of services. However to be sustainable, health interventions must be low cost and community-based. For example, medicine vendors and midwives, as well as women, could be trained to attend births and to provide basic care like malaria treatment and couples counselling. Pastors seem to play a central role in shaping the perceptions of communities about domestic violence, and thus, interventions to mitigate this social injustice against women should engage religious leaders and community elders to change the public view about this violence. They could also be involved in promoting mental health and suicide prevention awareness.

Other factors are also at play here as the prevalence of mental health issues, suicides, poverty, high birth rates, and housing issues are important psychosocial issues that worsen health outcomes for displaced women. Most of the problems of displacement, including violence against women in the home, malaria, HIV, and transactional sex, as well as a mental health crisis are all connected directly with income. Given this association, the economic empowerment of displaced communities should be of paramount concern at this time. Governments need to direct resources toward this aspect, recognizing that psychosocial health cannot respond easily to medical interventions if the socioeconomic causes of the health issues remains unaddressed. The unfortunate reality, however, is that the humanitarian need for health services far outweighs the available funding.

The results of this review suggest that policy interventions should focus on developing incentive-based home health intervention programs to significantly improve the knowledge, perception, and willingness of displaced women to take up available services. These include malaria prevention and care, mental health services, and hospital-based childbirth services. Mental health studies should also be merged with awareness and critical consciousness raising intervention activities. Comprehensive reproductive and sexual health services are also urgently needed.

## Figures and Tables

**Table 1:** Research articles on the health care of internally displaced women in Africa.

Author	Purpose of Study	Design	Country/Setting of Research	Study Results
Ager et al. (2018) [24]	To elicit local descriptions of gender-based violence experienced by women in camp	Participatory ranking method	Northern Uganda	Rape and intimate partner violence were of greatest concern. Normalization of violence within the home, where abusive actions were considered normal.
Stark et al. (2010) [25]	To establish incidence rates for gender-based violence in IDP camps in northern Uganda.	Interviews: a neighborhood methodology	Northern Uganda	Gender-based violence—particularly intimate partner violence—is commonplace in post-conflict Uganda
Stark et al. (2017) [26]	To identify discrepancies in the conceptualization and reporting on inter- personal violence in humanitarian settings.	Mixed method	DRC and Sudan	Group-based qualitative method elicited narratives of violence focusing on events perpetrated by strangers or members of the community more distantly connected to girls.
Cardoso et al. (2016) [27]	To understand the factors in the urban environment contributing to intimate partner violence experiences of women	Focus group	Cote d’Ivoire	Extreme financial insecurity and lack of social support predispose women to sexual exploitation when they try to access resources to provide for their families. The risk is higher for women without partners.
Aham-Chaibuotu et al. (2019) [28]	To examine the influence of conflict and displacement on gender relations, sexuality, and natality of internally displaced women in Nigeria	Focus group discussions and in-depth interviews	Northern Nigeria	Sexuality and procreation needed to be understood in the context of cultural values. The popular idea of sexual violence from a theoretical standpoint did not hold true for the examined population.
Logie et al. (2019) [29]	To explore factors associated with intimate partner violence and young adulthood violence among forcibly displaced young women	Cross-sectional survey	Kampala, Uganda	Sexual relationship was associated with decreased odds of poly-victmization. Normal actvities of daily living put young women at risk for sexual violation.
Oladeji et al. (2018) [30]	To report the disclosure and outcomes of sexual violence-related pregnancies (SVRP) among rescued female victims of Boko Haram insurgencies	Clinic records review	Borno state, Nigeria	The mean age of women with SVRP was 15 years. All concerned women desired to terminate their pregnancies but did not have access to abortion at the clinic because of the country’s abortion laws. Some were thought to have travelled outside the camp to have the abortion done.
Almedom et al. (2005) [31]	To assess the impact of prolonged displacement on the resilience of Eritrean mothers	Mixed methods	Eritrea	Women in camps had lower scores on Sense of Coherence compared with men.
Almedom et al. (2007) [32]	To identify the determinants of sense of coherence (resilience) in displaced Eritrean persons	Quantitative questionnaire approach: Sense of Coherence scale assessment	Eritrea: Northeast Africa	Displacement had a significantly negative effect on women compared with men. Hamboka women had the lowest Sense of Coherence score because of their experience of serial displacement.
Hamid et al. (2010) [33]	To investigate the effects of the Darfur crisis on the mental health of internally displaced women	Mixed methods	Darfur, Sudan	72% of the participants were classified as nonpsychotic psychiatric cases. Living conditions and security inside camps need improvement.
Kinyanda et al. (2010) [34]	To examine the long-term health consequences of war-related sexual violence among rural women living in camps	Purposive cross-sectional study design: structured interview	Northern Uganda	Age group of less than or equal to 44 years, being Catholic, and having at least one gynecologic complaint was connected with war-related sexual violence.
Olanrewaju et al. (2018) [35]	To explain the challenges of displacement and the coping startegies of internally diaplaced women in Nigeria	Qualitative approach with a descriptive survey	Yola and Abuja, northern Nigeria	Lack of social and financial support was a major challenge for women. Access to economic opportunities would affect coping.
Corbin et al. (2018) [36]	To explore resilience among internally displaced women in norther Uganda	Qualitative study	Nwoya and Gulu district, northern Uganda	Resilence was located in the women’s coping and maintenance of family and social relationships
Kim et al. (2007) [37]	To assess basic health, women’s health, and mental health among Sudanese IDPs in South Darfur	Questionnaire survey	Nyala Province, South Darfur, Sudan	Birth control use among IDP women was low and half of the population had experienced an unattended birth. The prevalence of major depression was 31%.
Kizza et al. (2012) [38]	To examine the role of alcohol in suicides	Qualitative psychological autopsy method	Northern Uganda	Economic disempowerment aggravated alcohol abuse in men, which had an effect on women’s mental health and suicide rates.
Kizza et al. (2012) [39]	To investigate suicide among women in a post-conflict context	Qualitative psychological autopsy interviews	Northern Uganda	The decision to choose suicide is linked to a pattern of unpleasant experiences that prevailed in the three months prior to the suicide.
Roberts et al. (2009) [40]	To measure the rates of post-traumatic stress disorder (PTSD) and depression among IDPs, and investigate associated demographic and trauma-exposure risk factors	Cross-sectional survey	Northern Uganda	18% of women and 8% of men had been raped or sexually abused. Gender was a determinant of mental distress, with women twice as likely as men to exhibit symptoms of PTSD and over four times as likely as men to exhibit symptoms of depression
Kisindja et al. (2017) [41]	To describe family planning awareness and needs among internally displaced women	Cross-sectional survey	DRC	Contraceptive knowledge among female camp residents was moderate, actual usage was low, and a considerable proportion reported a history of induced abortion, including self-induced abortion.
Ali et al. (2013) [42]	To investigate the unmet need for family planning and associated factors, and total demand for family planning	Community-based cross-sectional household survey	Eastern Sudan	Age, age at marriage, number of children, residence, and experience of child death were not associated with total unmet need for family planning. Housewives, and women with less than secondary education had higher total unmet need for family planning.
Decker et al. (2011) [43]	To assess the factors that influence the use of contraception among women in post-war Angola	Semi-structured interviews	Angola	Internally displaced women described difficulty paying for services, the lack of nearby services, and limited knowledge about contraceptive choices.
McGinn et al. (2011) [44]	To document and disseminate data on family planning knowledge, attitudes, and practices among displaced women	Population-based household surveys and health facility assessments	Sudan, Uganda, and the DRC	Use of modern contraceptive methods among women was under 4% in four program areas: West and South Darfur, Southern Sudan and Eastern Congo.
Orach et al. (2009) [45]	To explore female and male IDPs’ perceptions of their access to information about rights, access to health services, and experiences of gender-based violence	Cross-sectional study	Northern Uganda	Most women perceive gender-based violence as common in these settings. The main interventions include treatment of physical injuries, testing, treatment for STIs, and counselling.
Adam (2015) [46]	To determine the association between the place of delivery for maternal health education and home visits, and women’s socio-demographic characteristics	Cross-sectional study	Darfur-Sudan	Having home visits for maternal health education is associated with a 43% reduction in odds of giving birth at home, compared to not receiving home visits. The level of women’s education and the camp of residence predict home births.
Adam et al. (2015) [47]	To examine women’s awareness and use of reproductive health care services in emergency settings	Cross-sectional surveys	Darfur-Sudan	37% reported home-birth; 63% reported a facility-based delivery.
Chi et al. (2015) [48]	To explore perceptions of the effects of armed conflict on maternal and reproductive health services (MRH) and outcomes	Descriptive qualitative study	Burundi and northern Uganda	The perceived effects of the conflict on MRH outcomes include increased maternal and newborn morbidity and mortality; high prevalence of HIV/AIDS and SGBV; increased levels of prostitution, teenage pregnancy and clandestine abortion; and high fertility levels.
Kim et al. (2009) [49]	To analyze HIV, STI, and sexual risk as part of a larger reproductive health assessment of females in IDP camps	Two-stage random sample household survey	DRC	Sexually transmitted infection symptoms in the past 12 months and a history of sexual violence during the conflict were linked with HIV infection in the IDP population.
Obol et al. (2011) [50]	To assess the level of knowledge and misconception about malaria among pregnant women in post-conflict IDP camps	Cross-sectional study using a semi-structured questionnaire	Northern Uganda	Most pregnant women in the post-conflict IDP camps had knowledge about malaria symptoms but maintained misconceptions about the transmission and consequences.
Dræbel et al. (2013) [51]	To assess aspects of malaria infection, prevention, and treatment in a population of resettled pregnant women.	Cross-sectional study	South Sudan	Primary school attendance was a stronger predictor for use of malaria risk reduction measures than any of the other selected background characteristics.
Draebel et al. (2014) [52]	To explore lay perceptions of malaria and therapeutic process among 30 resettled pregnant women	Semi-structured interviews	South Sudan	Women relied on homemade remedies and concoctions, traditional healers’ cures, magical rituals, and private formal and informal medicine vendors at the local market before seeking a malaria diagnosis and treatment at the health center.
Obol et al. (2013) [53]	To establish the prevalence and factors associated with insecticide-treated net (ITN) use among pregnant women in IDP camps	Cross-sectional study	Northern Uganda	Factors that hinder ITN utilization were the hours taken to reach a health centre and being unmarried.
Brooks et al. (2017) [54]	To explore the factors influencing bednet ownership and use in an IDP camp with free bednet distribution	Mixed methods	Eastern Democratic Republic of Congo (DRC)	Health information on bednets was routinely provided in the camp, as noted by respondents. Some women who receive bednets resell them for money to purchase food items for their families.
